# Anti-Bacterial and Anti-Fouling Capabilities of Poly(3,4-Ethylenedioxythiophene) Derivative Nanohybrid Coatings on SUS316L Stainless Steel by Electrochemical Polymerization

**DOI:** 10.3390/polym12071467

**Published:** 2020-06-30

**Authors:** Chuan-Chih Hsu, Yu-Wei Cheng, Che-Chun Liu, Xin-Yao Peng, Ming-Chi Yung, Ting-Yu Liu

**Affiliations:** 1 Division of Cardiovascular Surgery, Department of Surgery, Taipei Medical University Hospital, Taipei Heart Institute, Taipei Medical University, Taipei 11031, Taiwan; cchsu1967@hotmail.com; 2 Department of Materials Engineering, Ming Chi University of Technology, New Taipei City 24301, Taiwan; liu820201@gmail.com (C.-C.L.); asd5997591@gmail.com (X.-Y.P.); 3 Department of Cardiovascular Surgery, Taiwan Adventist Hospital, and School of Medicine, National Yang Ming University, Taipei 105, Taiwan

**Keywords:** electrochemical polymerization, PEDOT, graphene oxide, anti-fouling capability, anti-bacterial capability

## Abstract

We have successfully fabricated poly(3,4-ethylenedioxythiophene) (PEDOT) derivative nanohybrid coatings on flexible SUS316L stainless steel by electrochemical polymerization, which can offer anti-fouling and anti-bacterial capabilities. PEDOT derivative nanohybrids were prepared from polystyrene sulfonates (PSS) and graphene oxide (GO) incorporated into a conducting polymer of PEDOT. Additionally, the negative charge of the PEDOT/GO substrate was further modified by poly-diallyldimethylammonium chloride (PDDA) to form a positively charged surface. These PEDOT derivative nanohybrid coatings could provide a straightforward means of controlling the surface energy, roughness, and charges with the addition of various derivatives in the electrochemical polymerization and electrostatically absorbed process. The characteristics of the PEDOT derivative nanohybrid coatings were evaluated by Raman spectroscopy, scanning electron microscopy (SEM), X-ray photoelectron spectroscopy (XPS), atomic force microscopy (AFM), water contact angle, and surface potential (zeta potential). The results show that PEDOT/PSS and PEDOT/GO nanohybrid coatings exhibit excellent anti-fouling capability. Only 0.1% of bacteria can be adhered on the surface due to the lower surface roughness and negative charge surface by PEDOT/PSS and PEDOT/GO modification. Furthermore, the anti-bacterial capability (7 mm of inhibition zone) was observed after adding PDDA on the PEDOT/GO substrates, suggesting that the positive charge of the PEDOT/GO/PDDA substrate can effectively kill bacteria (*Staphylococcus aureus*). Given their anti-fouling and anti-bacterial capabilities, PEDOT derivative nanohybrid coatings have the potential to be applied to biomedical devices such as cardiovascular stents and surgical apparatus.

## 1. Introduction

In recent decades, the development of human medical care has been paid increasing attention. In relation to this, films with anti-bacterial coatings have also been widely studied, including antibiotic materials, silver nanoparticles, copper nanoparticles, metal ions, and carbon nanotubes [1,2,3,4]. It is well-known that anti-bacterial materials use surface charges to improve anti-bacterial activities through the interaction with the different surface charge of the bacterial cell wall [5]. Generally, the wall of a bacterium with negative charge can be captured by anti-bacterial materials with positive charge, which can provide an excellent anti-bacterial effect [6]. Thus, the anti-bacterial activities of anti-bacterial materials can be improved by surface modifications of materials [7].

Poly(3,4-ethylenedioxythiophene) (PEDOT), a conducting conjugated polymer, has been widely used in various applications due to its excellent properties, including flexibility, chemical stability, high optical transparency, and good conductivity [8]. In addition, conducting conjugated PEDOT can be polymerized from EDOT monomers using different polymerization methods, such as chemical oxidative polymerization, vapor phase polymerization, and electrochemical polymerization [9,10,11]. Furthermore, conductive PEDOT-based derivatives, such as electrode films doped with poly(4-styrenesulfonate) (PSS) or different hydrophilic materials have attracted wide attention [12,13,14]. Due to good chemical and physical stability, the PEDOT/PSS can be used in a wide variety of applications such as organic solar cells, supercapacitors, antistatic surfaces, thermoelectric devices, and printed electronics [15,16]. Although oxidized PEDOT has excellent conductivity, it is unstable in water and solvents [17]. Therefore, the negative charge of PSS can be doped with the positive charge of conducting conjugated PEDOT to address this issue and improve its applicability.

In 2004, graphene nanosheets were successfully exfoliated from graphite using the process of repeatedly peeling flakes of Scotch tape [18]. Graphene of two-dimensional nanosheets with an atomic layer of carbon atoms has attracted significant scientific attention and technical interest due to its flexibility, chemical stability, high carrier mobility, and excellent electrical conductivity [19]. In addition, graphene oxide (GO) nanosheets were prepared by chemical modifications in which the surface of the GO exhibited a large number of functional groups (epoxide, carboxyl, carbonyl groups) containing oxygen. This promotes a negative charge of the surface to improve its dispersion in water [20]. These properties of GO-based nanosheets have provided great potential for wide applications in the biosensor, anti-bacterial, and optoelectronic devices [6,21,22]. Moreover, the surface negative charge of GO nanosheets can also be modified by the electrostatic absorbed method of positive charged poly-diallyldimethylammonium chloride (PDDA) to induce anti-bacterial capability [23]. In addition, the poor conductivity of GO nanosheets was improved to fabricate a novel conducting composite by a combination of conducting polymers [24,25,26]. Therefore, the PEDOT of conducting polymers has been incorporated in GO nanosheets to produce novel nanohybrid materials via self-assembly or chemical processes for electrochemical sensors and anti-fouling coatings [24,27,28].

In this study, PEDOT derivative nanohybrid coatings were fabricated using electrochemical polymerization on a flexible SUS316L stainless steel substrate. These PEDOT derivative nanohybrids were prepared by incorporation of a PSS dopant and GO nanosheets. In addition, the negatively charged PEDOT-GO nanohybrid coating was modified to form a positively charged surface of anti-bacterial film by electrostatically absorbed PDDA. The characteristics of the PEDOT derivative nanohybrid coatings were evaluated by Raman spectroscopy, scanning electron microscopy (SEM), X-ray photoelectron spectroscopy (XPS), atomic force microscopy (AFM), water contact angle, and surface potential (zeta potential). The anti-bacterial and anti-fouling capabilities of PEDOT derivative nanohybrid coatings were investigated by an inhibition zone and anti-fouling test. We anticipated that the PEDOT derivative nanohybrid coatings composed of GO nanosheets and PDDA would demonstrate the excellent anti-bacterial and anti-fouling capabilities, which can be applied to bio-interface coatings and biomedical devices.

## 2. Materials and Methods

### 2.1. Materials

SUS316L stainless steel substrate was purchased from Sinkang Industries Co., Ltd., (New Taipei City, Taiwan). Graphite powder was purchased from Allightec Co., (Taichung City, Taiwan). 3,4-Ethylenedioxythiophene (EDOT), PSS solution (Mw 75,000 g/mol), hydrogen peroxide (H_2_O_2_), hydrochloric acid (HCl), sulfuric acid (H_2_SO_4_), nitric acid (HNO_3_), sodium chloride (NaCl), alcohol, potassium permanganate (KMnO_4_), and PDDA solution were purchased from Sigma-Aldrich Corp. (St. Louis, MO, USA) and Acors Organics (Ozaukee County, WI, USA).

### 2.2. Preparation of GO Nanosheets

GO nanosheets were prepared from graphite powder by a modified Hummers-Offeman method [29]. First, 1 g of graphite powder was dispersed into 36 mL of H_2_SO_4_ solution for 30 min in an ice bath. Then, 12 mL of HNO_3_ solution and 5 g of KMnO_4_ were slowly added to the resulting solution, and the mixtures were stirred for 40 min in an ice bath. Subsequently, 120 mL of deionized (DI) water was gently added and stirred for 2 h. Then, 6 mL of H_2_O_2_ was added to the stirred solution for 2 h and settled overnight. After removing the supernatant, 200 mL of DI water, 1 mL of H_2_O_2_, and 1 mL of HCl were added to above resultant mixture and stirred for 2 h. The resultant solution was washed and centrifuged three times. The resultant was washed with DI water until a neutral pH was achieved to obtain dark-yellow GO nanosheets, which were dried by vacuum freeze drying for 72 h.

### 2.3. Preparation of PEDOT/PSS and PEDOT/GO Nanohybrid Coatings

A PEDOT/PSS nanohybrid coating was prepared from an EDOT monomer with PSS and GO nanosheets by electrochemical polymerization on a SUS316L stainless steel substrate. The SUS316L stainless steel substrate was washed sequentially with common detergent, ethanol, and DI water, which was dried in a vacuum oven. First, 56.8 μL of EDOT and 113.6 μL of PSS were dispersed into DI water and ultrasonicated for 2 h at room temperature. Then, the solution was placed into a three-electrode electrochemical analyzer (PGSTAT12, Metrohm Autolab B.V., Utrecht, Netherlands) and polymerized at a constant potential of 1 V to fabricate a PEDOT/PSS nanohybrid coating with an average deposition density of 10–20 mC cm^−2^. The electrochemical polymerization was performed using a three-electrode electrochemical analyzer, including the reference electrode of AgCl, the counter electrode of Pt wire, and the working electrode of a SUS316L stainless steel substrate. According to the above procedure, 56.8 μL of EDOT and 0.1 g of GO nanosheets were dispersed into DI water and ultrasonicated for 2 h. Then, the above solution was polymerized on SUS316L stainless steel substrate to obtain a PEDOT/GO nanohybrid coating by electrochemical polymerization.

### 2.4. Preparation of PEDOT/GO/PDDA Nanohybrid Coating

**Scheme 1:**
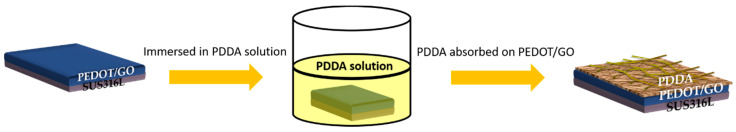
Preparation of the poly(3,4-ethylenedioxythiophene)/graphene oxide/poly-diallyldimethylammonium chloride (PEDOT/GO/PDDA) nanohybrid coating.

PEDOT/GO/PDDA nanohybrid coating was prepared from PEDOT/GO and PDDA by the electrostatic absorbed method, as illustrated in Scheme 1. The PEDOT/GO nanohybrid coating was immersed into PDDA solution for 30 min. Then, the coating was washed with DI water three times. The resultant PEDOT/GO/PDDA nanohybrid coating was dried at 60 °C.

### 2.5. Anti-Bacterial Capability of PEDOT Derivatives Nanohybrid Coatings

The anti-bacterial capability of PEDOT derivative nanohybrid coatings was surveyed in connection with bacteria (*Staphylococcus aureus*, *S. aureus*). For the microbiological experiment, all of the chemicals were autoclaved at 120 °C for 15 min. *S. aureus* bacteria were incubated at 37 °C for 18–24 h. The cultured *S. aureus* was mixed with 1 wt % NaCl solution and stirred in a vortex mixer for 30 s. Then, 1 mL of the solution was added to 9 mL of Lysogeny broth (LB) medium and stirred in a vortex mixer for 30 s. According to the above procedure, the mixing process was repeated three times. Subsequently, 0.01 mL of suspension *S. aureus* (10^5^ CFU/mL) was dropped and uniformly dispersed on a nutrient agar plate. The PEDOT derivative nanohybrid coatings were placed on the nutrient agar plate with *S. aureus* and incubated at 37 °C for 18–24 h. After 18–24 h of culturing, the inhibition zone of PEDOT derivative nanohybrid coatings was demonstrated.

### 2.6. Anti-Fouling Capability of PEDOT Derivatives Nanohybrid Coatings

A quantity of 10^5^ CFU/mL of *S. aureus* was chosen as the adhering bacteria for the evaluation of anti-fouling capability. The PEDOT derivative nanohybrid coatings were immersed in the *S. aureus* solution and incubated at 37 °C for 18–24 h. The number of *S. aureus* bacteria adsorbed on the PEDOT derivative nanohybrid coatings was counted by fluorescence microscope (fluorescent nucleic acid stain-SYTO^®^9/propidium iodide kits).

### 2.7. Characterization

Raman measurement with a 532 nm He-Ne emitting laser was performed using a micro-Raman system (Renishaw Vendor, Gloucestershire, UK) in the detection range from 1000 to 3000 cm^−1^. The chemical binding conditions of PEDOT derivative nanohybrid coatings were measured by X-ray photoelectron spectrometer (XPS, ULVAC-PHI PHI 5000 VersaProbe, Ulvac-PHI, Kanagawa, Japan). The surface charge of PEDOT derivative nanohybrid coatings was observed by zeta potential (Zetasizer 3000, Malvern Instruments, Malvern, UK). A contact angle goniometer (DSA 100, Krüss GmbH, Hamburg, Germany) was used for the static contact angle measurement of PEDOT derivative nanohybrid coatings. An atomic force microscope (AFM, Dimension Edge, Bruker, Madison, WI, USA) and field emission scanning electron microscope (FE-SEM, JEOL JSM 6701F, JEOL Co., Tokyo, Japan) were used for the morphological characterization of PEDOT derivative nanohybrid coatings. The number of bacteria was calculated from fluorescence microscope images bacteria adhered on the surface of the SUS316L substrate and PEDOT derivative nanohybrid coatings by using commercial ImageJ software (National Institutes of Health, Bethesda, MD, USA).

## 3. Results and Discussion

### 3.1. Characteristics of PEDOT Derivative Nanohybrid Coatings

**Figure 1:**
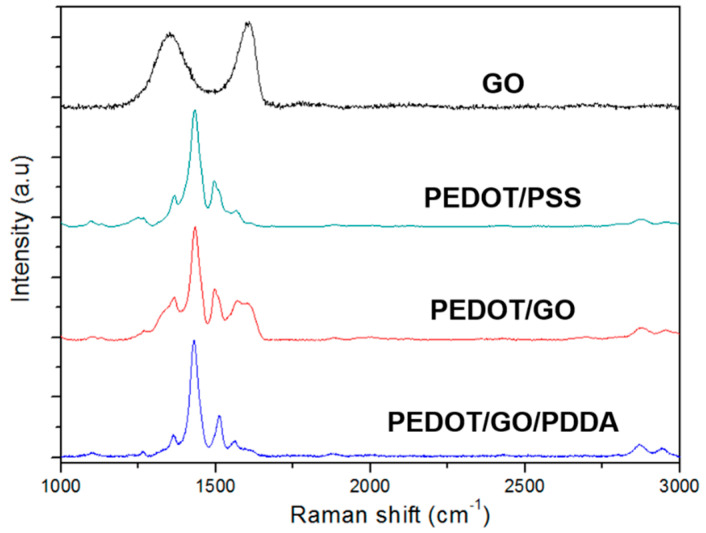
Raman spectra of GO nanosheets, and PEDOT/PSS (polystyrene sulfonates), PEDOT/GO, and PEDOT/GO/PDDA nanohybrid coated substrates.

The structural properties of GO nanosheets and PEDOT derivative nanohybrid coatings were measured by Raman spectroscopy, as shown in Figure 1. The GO nanosheets exhibited the characteristic peaks at 1350 cm^−1^ (D band) for the in-plane bond stretching of sp^2^ carbon atoms and 1583 cm^−1^ (G band) for defects of structure and lattice distortion [30]. The Raman spectrum of the PEDOT/PSS nanohybrid coating was observed at 1433 and 1505 cm^−1^ for the asymmetric and symmetric C=C stretching vibration of PEDOT [31]. When the GO nanosheets were used in the PEDOT nanohybrid system, the Raman spectrum of the PEDOT/GO nanohybrid coating was similar to that of the PEDOT/PSS nanohybrid coating, while the shoulder peaks of the D and G bands of the GO nanosheets were located at 1350 and 1583 cm^−1^, respectively. This confirms that GO nanosheets were doped in the chain [32]. After the electrostatically absorbed process by PDDA, the Raman spectrum of the PEDOT/GO/PDDA nanohybrid coating was similar to that of the PEDOT/GO nanohybrid coating.

**Figure 2:**
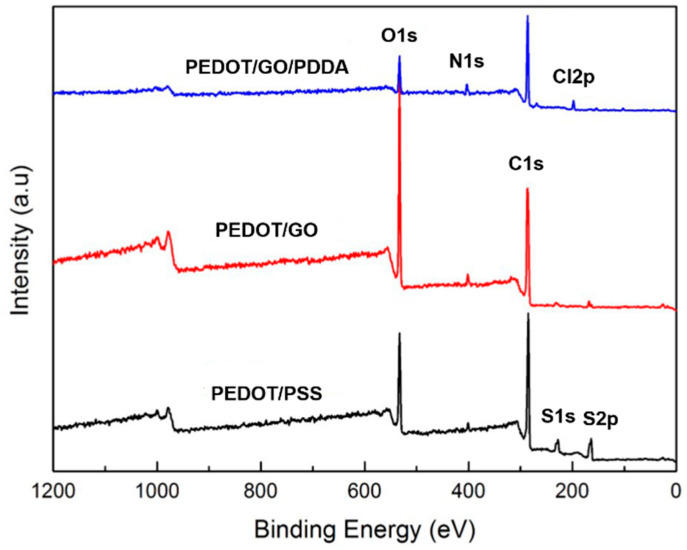
X-ray photoelectron spectroscopy (XPS) survey spectra of PEDOT/PSS, PEDOT/GO, and PEDOT/GO/PDDA nanohybrid coatings.

**Figure 3:**
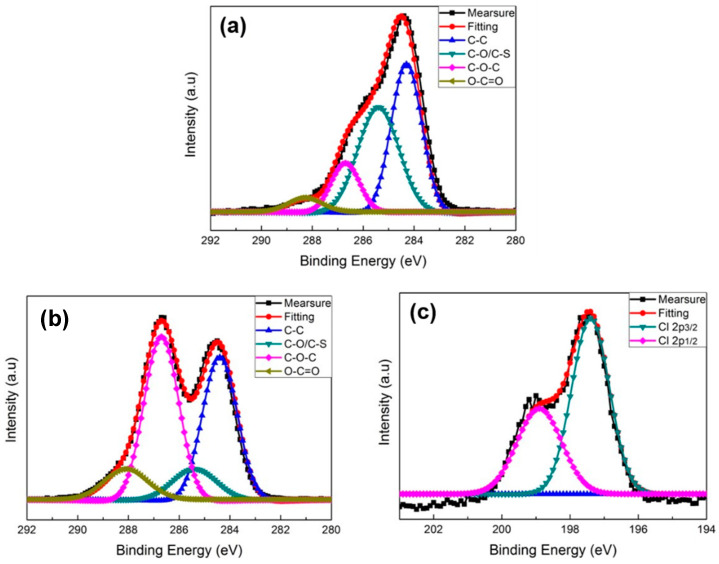
C-1s XPS spectra of (**a**) PEDOT/PSS and (**b**) PEDOT/GO nanohybrid coatings, and (**c**) Cl-2p spectra of PEDOT/GO/PDDA nanohybrid coated substrates.

The chemical binding of PEDOT derivative nanohybrid coatings was analyzed by XPS spectra, as shown in Figure 2. The XPS spectra were observed in the presence of oxygen (O-1s), carbon (C-1s), and sulfur (S-2s and S-2p) elements in the PEDOT/PSS nanohybrid coating. Compared with the PEDOT/PSS nanohybrid coating, the characteristic peaks (S-2s and S-2p) of the PEDOT/GO nanohybrid coating were almost dispersed, while the O-1s peak was increased with oxygen-containing groups of GO nanosheets incorporated into the PEDOT nanohybrid system. Moreover, a new characteristic peak (Cl-2p) was observed for PEDOT/GO/PDDA nanohybrid coatings, indicating that PDDA was absorbed on the PEDOT/GO nanohybrid coating by the electrostatic absorbed process. The curve-fitting of C-1s spectra of PEDOT/PSS and PEDOT/GO nanohybrid coatings are shown in Figure 3a,b. Compared with the PEDOT/PSS nanohybrid coating, the stronger characteristic peak at 286.8 eV for the C-O-C bond was observed for the PEDOT/GO nanohybrid coating. The results indicate that the many oxygen-containing functional groups of GO nanosheets were successfully incorporated into the PEDOT nanohybrid system [31]. In addition, the curve-fitting of Cl-2p spectra (Figure 3c) of PEDOT/GO/PDDA nanohybrid coatings demonstrates characteristic peaks at 198.8 and 197.4 eV, corresponding to Cl-2p_1/2_ and Cl-2p_3/2_, which further confirm the adsorption of PDDA on the PEDOT/GO nanohybrid coating.

**Figure 4:**
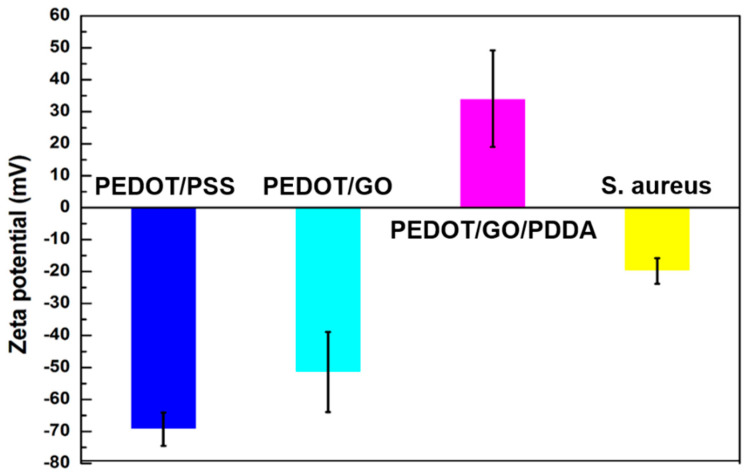
Zeta potential values of nanohybrid coated substrates (PEDOT/PSS, PEDOT/GO, PEDOT/GO/PDDA substrates) and bacteria (*S. aureus*).

The zeta potential results of the nanohybrid coated substrates (PEDOT/PSS, PEDOT/GO, PEDOT/GO/PDDA substrates) and bacteria (*S. aureus*) are illustrated in Figure 4. Due to the negative charge of PSS and GO nanosheets incorporated into the PEDOT nanohybrid system, the zeta potential values of PEDOT/PSS and PEDOT/GO nanohybrid coatings were −69.3 and −51.4 mV, respectively. When positively charged PDDA was absorbed on PEDOT/GO nanohybrid coating, the zeta potential value of PEDOT/GO/PDDA substrate significantly increased to +34.1 mV. In addition, the zeta potential value of *S. aureus* with the negative charge of the cell walls was −19.8 mV. Therefore, the positive charge of PEDOT/GO/PDDA substrate can physically absorb and thus kill the bacteria (*S. aureus*), thereby inducing the anti-bacterial capability.

**Figure 5:**
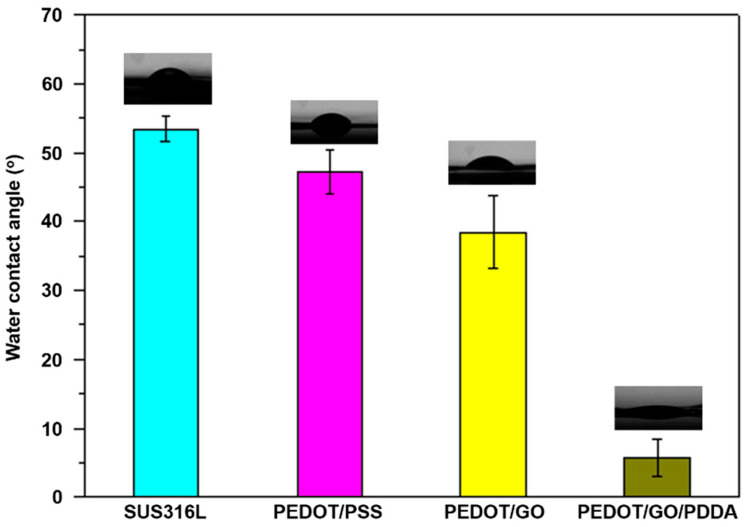
Water contact angle of the SUS316L substrate, and PEDOT/PSS, PEDOT/GO and PEDOT/GO/PDDA nanohybrid coatings.

The surface hydrophilicity of PEDOT derivative nanohybrids coated on SUS316L stainless steel substrate was investigated by water contact angle measurement, as shown in Figure 5. The surface of pristine SUS316L substrate displayed a water contact angle of 53.4°. After the PEDOT/PSS nanohybrid was coated on the SUS316L substrate, the water contact angle of the coated surface decreased to 47.2°. Moreover, the water contact angle (38.4°) of the PEDOT/GO nanohybrid coating was slightly lower than that of the PEDOT/PSS nanohybrid coating, indicating that the incorporation of GO nanosheets possessed a large number of oxygen functional groups. Furthermore, the PEDOT/GO/PDDA nanohybrid coating demonstrated the lowest water contact angle (5.7°). The results indicate the successful adsorption of hydrophilic PDDA on the PEDOT/GO nanohybrid substrate to create the hydrophilic surface.

**Figure 6:**
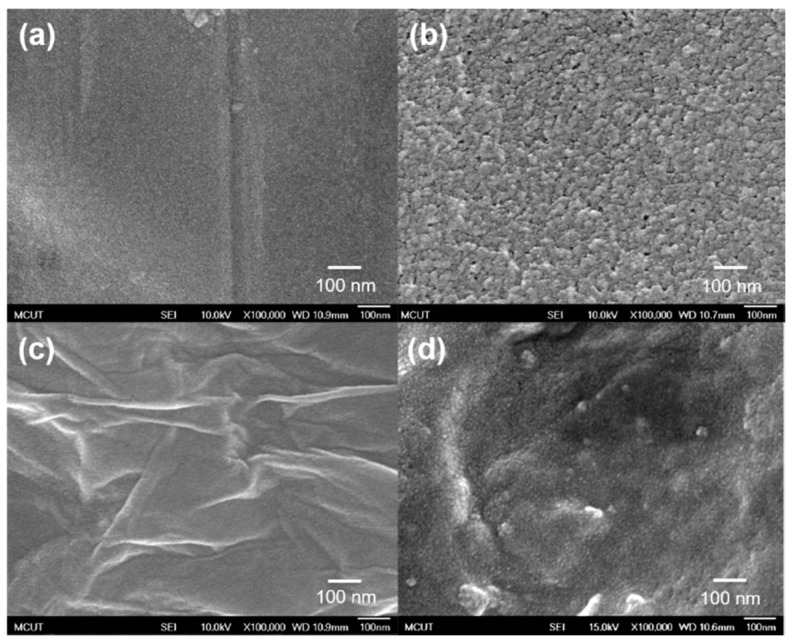
Top-view SEM images of (**a**) the pristine SUS316L stainless steel substrate, and (**b**) PEDOT/PSS, (**c**) PEDOT/GO, and (**d**) PEDOT/GO/PDDA modified SUS316 substrates.

The surface morphological characterization of the SUS316L substrate and PEDOT derivative nanohybrid coatings were investigated by SEM, as shown in Figure 6. Macroscopically, the pristine SUS316L stainless steel substrate showed a clear and smooth surface morphology (Figure 6a). The PEDOT/PSS nanohybrid coating was observed as a dense layer on the SUS316L substrate, as shown in Figure 6b. Moreover, the PEDOT/GO nanohybrid coating was shown as GO nanosheets with a wrinkled structure on the surface of the PEDOT nanohybrid system (Figure 6c). When the positively charged PDDA was absorbed on the PEDOT/GO nanohybrid coating (Figure 6d), the absence of GO nanosheets with wrinkled morphology on the surface of PEDOT/GO/PDDA nanohybrid coatings indicated that PDDA successfully covered the surface of the PEDOT/GO nanohybrid coated substrate. The surface roughness of coating layers was further investigated by AFM analysis.

**Figure 7:**
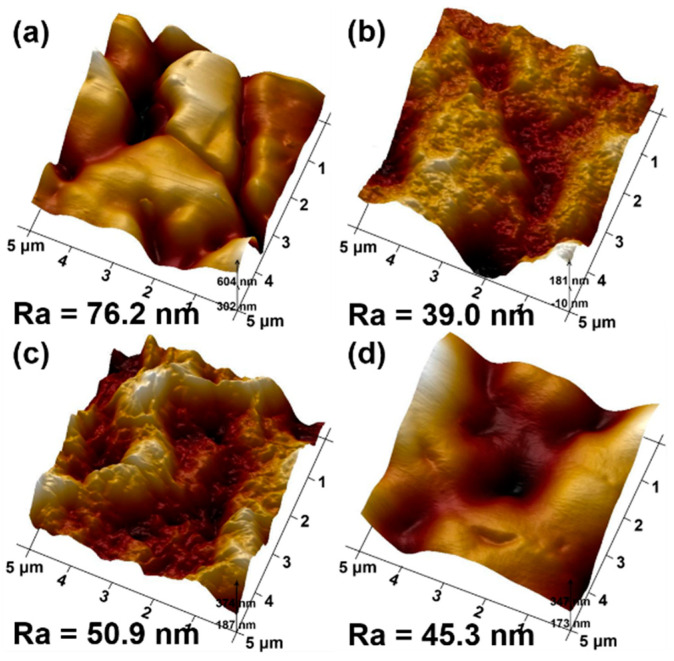
Atomic force microscopy (AFM) images of (**a**) the SUS316L substrate, and (**b**) PEDOT/PSS, (**c**) PEDOT/GO, and (**d**) PEDOT/GO/PDDA nanohybrid coatings.

Figure 7 shows the microscopic surface roughness and morphology of the SUS316L substrate and PEDOT derivative nanohybrid coatings by AFM. Microscopically, the pristine SUS316L substrate displayed a relatively rough morphology (Ra = 76.2 nm). By the electrochemical polymerization process, the PEDOT/PSS nanohybrid was polymerized and filled the nanostructured surface of SUS316L substrate to obtain a uniform surface morphology and lower surface roughness (Ra = 39.0 nm). Compared with the PEDOT/PSS nanohybrid coating, the surface roughness of the PEDOT/GO nanohybrid coating increased from 39.0 to 50.9 nm, which was due to the presence of GO nanosheets on the surface. When positively charged PDDA was absorbed on the PEDOT/GO nanohybrid coating, the surface roughness of the PEDOT/GO/PDDA nanohybrid coating decreased to 45.3 nm. The result shows that the PDDA covered the surface, reducing surface roughness and creating a relatively smooth morphology.

### 3.2. Anti-Fouling Capability of PEDOT Derivative Nanohybrid Coatings

**Figure 8:**
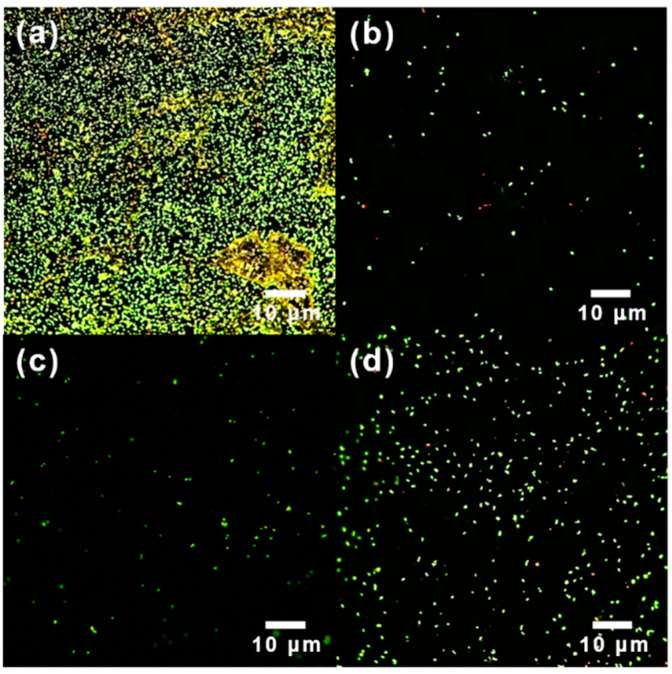
The number of bacteria adhering to (**a**) the SUS316L substrate, and (**b**) PEDOT/PSS, (**c**) PEDOT/GO, and (**d**) PEDOT/GO/PDDA nanohybrid coatings.

To evaluate the anti-fouling capability of the PEDOT derivative nanohybrid coated on the surface of SUS316L stainless steel (Figure 8), 10^5^ CFU/mL of S. aureus was chosen as the model bacteria. Green dots of the fluorescence microscope images represent live *S. aureus* bacteria adhering to the surface of the SUS316L substrate and PEDOT derivative nanohybrid coatings. A dense distribution of *S. aureus* (approximately 10^8^/cm^2^) adhered to the surface of the pristine SUS316L substrate, as shown in Figure 8a. Compared with the pristine SUS316 substrate (Ra = 76.2 nm), a lower adhesion density of bacteria was obtained on the PEDOT derivative nanohybrid coatings (Figure 8b–d), which was due to the lower surface roughness. Therefore, the numbers of adhered bacteria were decreased to approximately 10^6^/cm^2^ for PEDOT/PSS, approximately 10^6^/cm^2^ for PEDOT/GO, and approximately 10^7^/cm^2^ for PEDOT/GO/PDDA substrates. In particular, the negative charge of PEDOT/PSS (Figure 8b) and PEDOT/GO (Figure 8c) nanohybrid coatings can further inhibit and reduce the adhesion of the negative charge of *S. aureus*, demonstrating the anti-fouling capability. On the other hand, the numbers of *S. aureus* decrease from 10^5^ CFU/mL (SUS316L substrate) to approximately 10^2^ CFU/mL (PEDOT/PSS and PEDOT/GO nanohybrid coating), and only 0.1% of bacteria can be adhered on the surface. However, the numbers of adhering bacteria (approximately 10^3^ CFU/mL) on the PEDOT/GO/PDDA nanohybrid coating were slightly higher than that of the PEDOT/PSS and PEDOT/GO nanohybrid coating. The results indicate that the bacteria seemed to slightly prefer to adhere on the positive charge of the PEDOT/GO/PDDA substrate (Figure 8d). The negative cell wall of the bacteria would be locked by the positive charge of PEDOT/GO/PDDA substrates to inhibit the growth; thus, the bacteria are going to die [6]. The substrate exhibited excellent anti-bacterial capability, as discussed in the next section.

### 3.3. Anti-Bacterial Capability of PEDOT Derivative Nanohybrid Coatings

**Figure 9:**
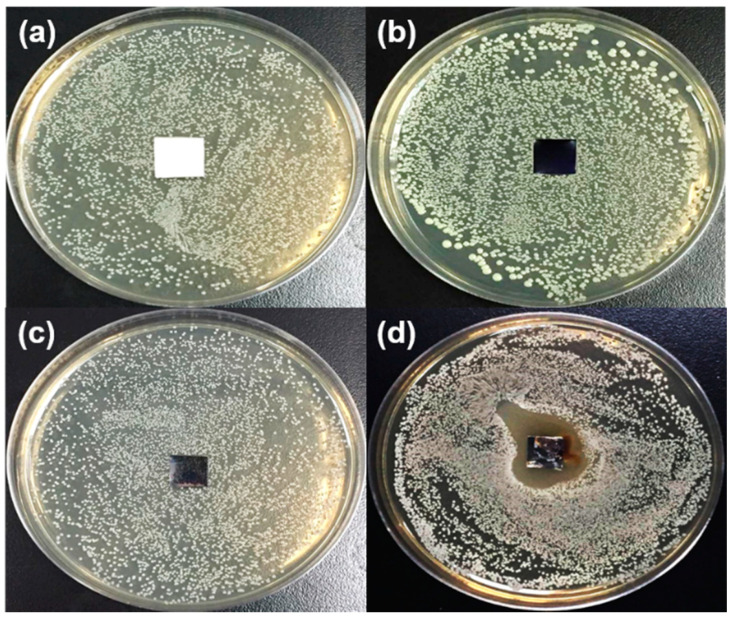
Anti-bacterial capability (inhibition zone) of (**a**) the SUS316L substrate, and (**b**) PEDOT/PSS, (**c**) PEDOT/GO, and (**d**) PEDOT/GO/PDDA substrates.

The anti-bacterial capability of PEDOT derivative nanohybrid coatings was evaluated using *S. aureus* as a model bacterium. As compared with other anti-bacterial tests, the zone of inhibition testing is a rapid and inexpensive test for anti-bacterial activity. Nevertheless, the zone of inhibition testing is a qualitative test to inhibit the growth of tested bacteria. In this study, the anti-bacterial capability was investigated by measuring the inhibition zone incubated on a nutrient agar plate, as shown in Figure 9. When the zone of inhibition shows on the nutrient agar plate, the results displayed that the tested bacteria were susceptible to the PEDOT derivative nanohybrid coatings. The pristine SUS316L substrate, and the PEDOT/PSS and PEDOT/GO nanohybrid coatings, showed the absence of an inhibition zone. However, a significant inhibition zone (7 mm) on the PEDOT/GO/PDDA nanohybrid coating was observed. This is due to the positive charge of PDDA absorbed on the PEDOT/GO substrate to create the positive charge surface of the PEDOT/GO/PDDA substrate, which can inhibit the growth of the bacteria. Compared with our previous studies [24,33], the negative charge of PEDOT derivatives (PEDOT/PSS, PEDOT/GO, PEDOT/Heparin, PEDOT/ chondroitin sulfate, and PEDOT/carboxymethyl-hexanoyl chitosan) can suppress the adhesion of the negative charge of proteins, platelets, and bacteria to form the anti-fouling surface. However, it would be an anti-bacterial surface after immobilizing the positive charge of PDDA on the PEDOT/GO substrate. Therefore, the inclusion of PDDA in the PEDOT/GO/PDDA substrate can effectively diffuse and then kill bacteria, as shown as the larger inhibition zone [6].

## 4. Conclusions

This study successfully fabricated PEDOT derivative nanohybrid coatings on a flexible SUS316L stainless steel substrate by electrochemical polymerization for improving the anti-bacterial and anti-fouling capabilities. Via the addition of hydrophilic derivatives, these PEDOT derivative nanohybrid coatings decrease the water contact angle and surface roughness to form a hydrophilic surface. In addition, the negatively charged surface of the PEDOT/GO nanohybrid coating can be further modified by the electrostatically absorbed process of the positively charged PDDA. PEDOT/PSS and PEDOT/GO nanohybrid coatings with lower surface roughness and negative charge led to less adsorption of bacteria (*S. aureus*). By comparison, the positively charged PEDOT/GO/PDDA nanohybrid coating inhibited the growth of bacteria, resulting in excellent anti-bacterial capability. Therefore, the electrochemically polymerized PEDOT derivative nanohybrid coatings can provide an anti-fouling and anti-bacterial surface, which offers a straightforward and rapid method to develop anti-bacterial and anti-fouling coatings for medical devices.
